# The anti-cancer effects of phenformin in thyroid cancer cell lines and in normal thyrocytes

**DOI:** 10.18632/oncotarget.27266

**Published:** 2019-11-05

**Authors:** Francesca Coperchini, Laura Croce, Marco Denegri, Oriana Awwad, Samuel Tata Ngnitejeu, Flavia Magri, Luca Chiovato, Mario Rotondi

**Affiliations:** ^1^ Istituti Clinici Scientifici Maugeri IRCCS, Unit of Internal Medicine and Endocrinology, Laboratory for Endocrine Disruptors, Pavia, Italy; ^2^ PHD course in Experimental Medicine, University of Pavia, Pavia, Italy; ^3^ Molecular Cardiology, ICS-Maugeri, Pavia, Italy; ^4^ Department of Biopharmaceutics and Clinical Pharmacy, The University of Jordan, Amman, Jordan; ^5^ Department of General and Minimally Invasive Surgery, Istituti Clinici Scientifici Maugeri IRCCS, Pavia, Italy; ^6^ Department of Internal Medicine and Therapeutics, University of Pavia, Pavia, Italy

**Keywords:** phenformin, tumor-microenvironment, thyroid cancer, CXCL8

## Abstract

Phenformin is a biguanide drug which, besides the original anti-diabetic effect, also exerts anti-cancer effects. The aim of this study was to further characterize these latter in terms of both cell-viability and modulation of the secretion of the pro-tumorigenic chemokine CXCL8. Normal human thyrocytes in primary cultures (NHT) and thyroid cancer cell lines, TPC-1 and 8505C (RET/PTC and BRAFV600E mutated, respectively) were treated with increasing concentrations of phenformin at different times. Cell-viability was assessed by WST-1 and further characterized by AnnexinV/PI staining and cell proliferation colony-assay. CXCL8 levels were measured in cell supernatants. Phenformin reduced cell-viability in TPC-1 and 8505C and their ability to form colonies. In NHT cells, phenformin affected cell-viability only at the maximal dose but interestingly it inhibited CXCL8 secretion at all the concentrations not affecting cell-viability. Phenformin had no effect on CXCL8 secretion in thyroid cancer cell lines. Thus, phenformin exerts anti-cancer effects on both cancer cells (cell death induction) and surrounding normal cells (inhibition of CXCL8 secretion). These results highlight that the anti-cancer effects of phenformin are multifaceted and effective on both solid and soluble components of the tumor-microenvironment.

## INTRODUCTION

Thyroid cancer is the most prevalent endocrine malignancy with an overall increasing rate of 3% annually [1]. Surgery is the definitive treatment option for most papillary thyroid cancers (PTCs), followed by radioiodine (RAI) therapy in selected patients. However, a minority of patients with PTCs (5–10%) develop recurrence and distant metastasis and fail to respond to RAI therapy [2]. These treatment-resistant patients still represent a clinical challenge; thus, the development of new therapeutic strategies aimed at blocking thyroid cancer progression are highly required. The anti-diabetic biguanide drugs (e.g. metformin and phenformin) were reported to also have anti-cancer effects [3, 4]. Since many years, phenformin was banned as an anti-diabetic drug due to the occurrence of a major side effect such as lactic acidosis [5]. Meanwhile, phenformin was shown to have some anti-cancer activities in several types of tumors including cholangiocarcinoma [6]; ovary [7], breast [8], rectal [9], brain [10] and pituitary [11] cancers; melanoma [12], and neuroendocrine cancers [13]. Like metformin, phenformin is a mitochondrial complex I inhibitor [14, 15], but, due to its hydrophobic moiety, it is transported with a greater affinity and a more rapid kinetic into cells [16]. Experimental data support the view that the anti-neoplastic effects of phenformin are stronger than those of metformin [17], and include: inhibition of cell proliferation [18, 19]; induction of neoplastic cell apoptosis [20, 21]; *in vivo* suppression of tumor development and growth [10, 20, 22, 23]; inhibition of mesenchymal-epithelial transition [8]; and inhibition of angiogenesis [24]. Interestingly, a recent study in melanoma demonstrated that phenformin enhances the effects resulting from anti-PD-1 immune checkpoint blockade, thus suggesting a new anti-cancer effect of the drug [12]. This effect specifically occurred in infiltrating immune cells, a major component of the so called “tumour microenvironment”, which is composed not only by normal and cancer cells, but also by cells and soluble mediators (chemokines) of the immune system [25, 26]. Phenformin is currently tested in a phase I trial aimed at identifying the optimal dose for a combined treatment with small molecule targeted drugs (Dabrafenib and Trametinib) in patients with BRAF mutated melanoma (NCT03026517).

With specific regard to thyroid cancer, metformin was found to reduce cell proliferation [26], to inhibit the secretion of the pro-tumorigenic chemokine CXCL8 [27], and to induce thyroid cancer cell death [28]. No studies so far evaluated the effects of phenformin in thyroid cancer. Aim of the present study was to investigate the potential anti-cancer effect of phenformin in terms of cell viability and modulation of CXCL8 secretion in normal and thyroid cancer cells.

## RESULTS

### Effect of phenformin on NHT, TPC-1 and 8505C thyroid cells viability

**Figure 1:**
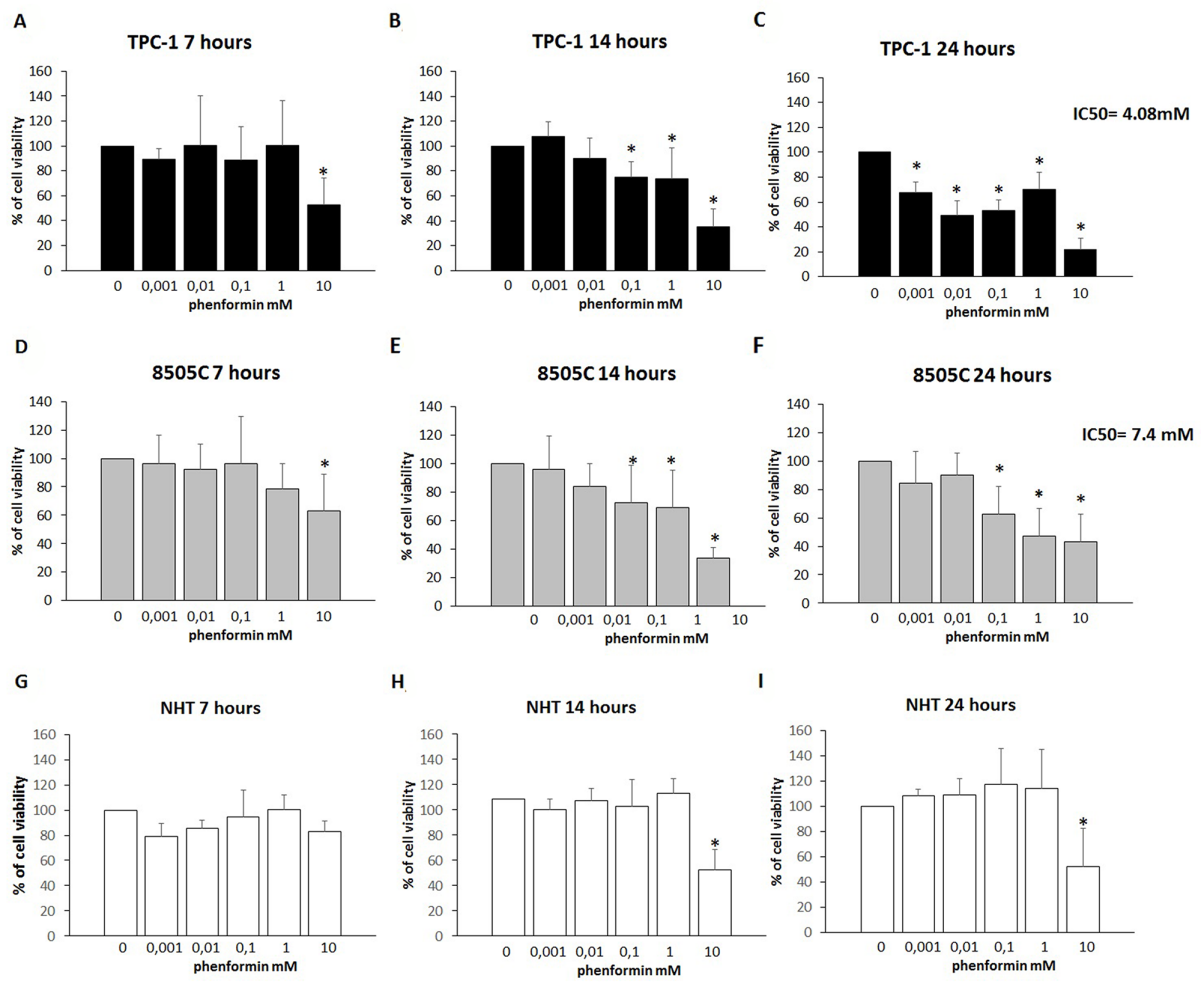
Cell viability of TPC-1, 8505C and NHT after treatment with Phenformin at increasing concentrations at different time points **Panel A)** Phenformin 10mM reduces TPC-1 viability after 7 hours of cell (ANOVA F=3.765; p<0.005). **Panel B)** Phenformin 10 mM reduces cell viability after 14 hours of cell incubation (ANOVA F=12.160; p<0.001). **Panel C)** Phenformin reduces cell viability at all concentration tested after 24 hours of cell incubation (ANOVA F=42.537; p<0.001). **Panel D)** Phenformin 10mM reduces 8505C viability after 7 hours of cell (ANOVA F=3.482; p<0.05). **Panel E)** Phenformin reduces cell viability after 14 hours of cell incubation starting from 0.1 mM (ANOVA F=15.007; p<0.001). **Panel F)** Phenformin reduces cell viability after 24 hours of cell incubation starting from 0.1 mM (ANOVA F=10.129; p<0.001). **Panel G)** Phenformin does not affect NHT cells viability after 7 hours (ANOVA NS). **Panel H)** Phenformin 10 mM reduces cell viability after 14 hours of cell incubation (ANOVA: F=8.892: p<0.001). **Panel I)** Phenformin reduces cell viability at all concentration tested after 24 hours of cell incubation (ANOVA F=12.7; p<0.001). IC50 values are shown for 24h treatment with phenformin of TPC-1 and 8505C. ^*^Post Hoc analysis by Bonferroni p<0.05 vs. basal.

To assess changes in thyroid cells viability, a time-course incubation experiment was performed. Cells were incubated for 7, 14 and 24 hours in the presence of increasing concentrations of phenformin. As shown in Figure 1 (Panel A-B-C), treatment with phenformin reduced TPC-1 cell viability in a time- and dose-dependent manner. Incubation with 10 mM phenformin reduced cell viability after 7 hours (ANOVA F=3.765; p<0.005; Post Hoc 10mM p<0.05 vs. basal) (Figure 1 Panel A). A more pronounced effect on TPC-1 cell viability was observed after a longer exposure time even at lower concentrations of phenformin. Significant reduction of TPC1 cell viability was observed starting from 0.1 mM concentration (ANOVA F=21.664; p<0.001; Post Hoc 0.1, 1 and 10 mM p<0.05 vs. basal) (Figure 1 Panel B) after 14 hours and starting from 0.001 mM after 24 hours (ANOVA F=42.537; p<0.001; Post Hoc all concentrations p<0.05 vs. basal) (Figure 1 Panel C). Similarly, in 8505C, phenformin reduced cell viability starting from a 7-hour incubation time but only at the maximal concentration of 10 mM (ANOVA F=3.482; p<0.05; Post Hoc 10 mM p<0.05 vs. basal) (Figure 1 panel D). Significant reduction of 8505C cell viability was observed starting from a 0.1 mM concentration after 14 hours (ANOVA F=15.007; p<0.001; Post Hoc 0.1, 1 and 10 mM p<0.05 vs. basal) (Figure 1 Panel E) and after 24 hour of treatment (ANOVA F=10.129; p<0.001; Post Hoc 0.1, 1 and 10 mM p<0.05 vs. basal) (Figure 1 Panel F). Unlike thyroid cancer cells, phenformin did not reduce viability in NHT cells after a 7 hour incubation time at any of the used concentrations (ANOVA: F=1.865; NS) (Figure 1 Panel G). A reduction of NHT cells viability was observed only at the maximal concentration of phenformin (10 mM) after 14 (ANOVA: F=8.892: p<0.001; *Post Hoc* 10mM p<0.05 vs. basal) and 24 (ANOVA F=12.7; p<0.001; *Post Hoc* 10mM p<0.05 *vs*. basal) (Figure 1 Panel H-I) hours of incubation.

**Figure 2:**
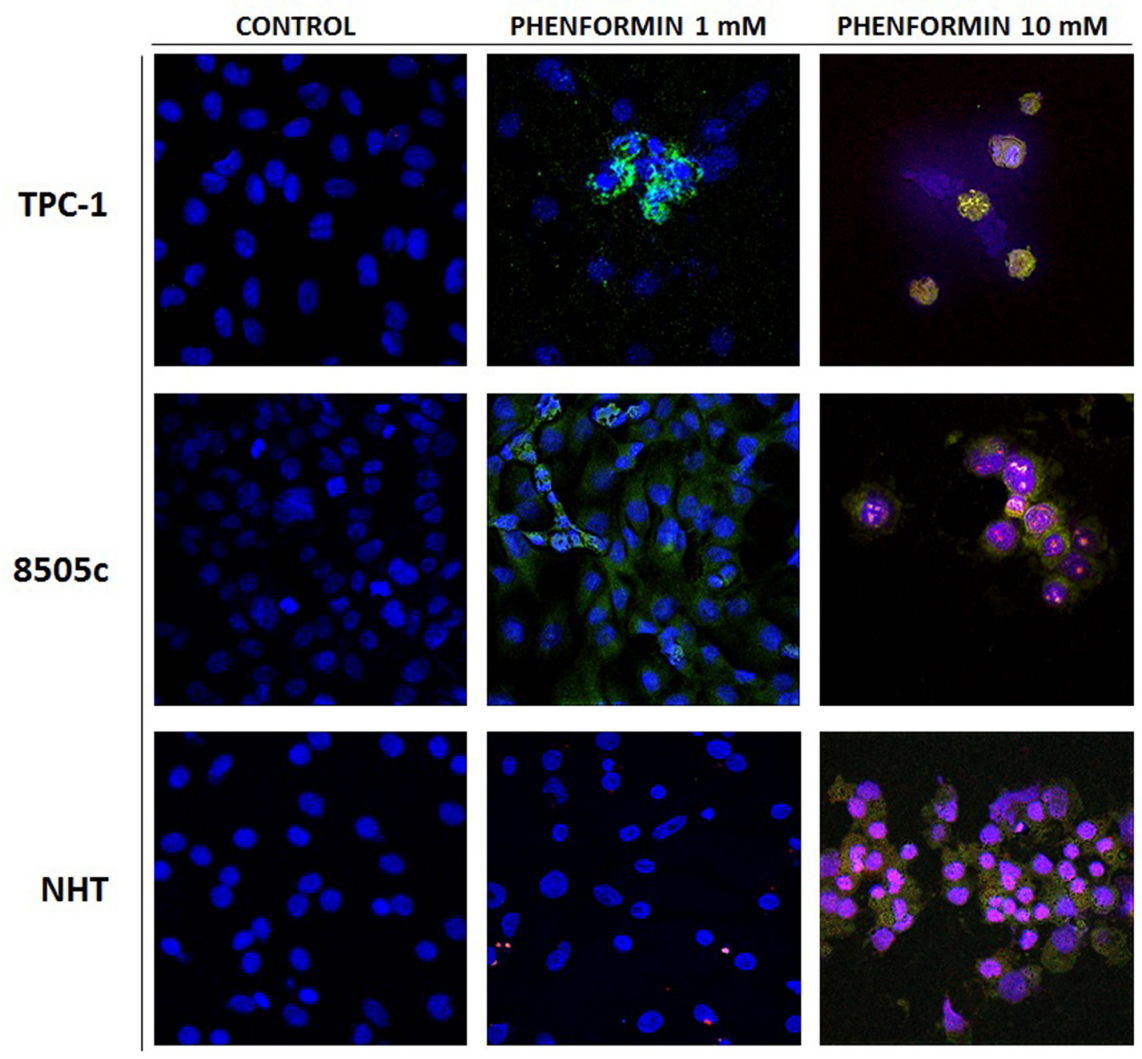
Apoptosis induced in treated thyroid cancer cell lines observed with Annexin V-FITC/PI stain under confocal fluorescent microscope (40× magnification) These are representative of merged Annexin V/PI/Hoecst images of three experiments. The control untreated cells appear positive only for hoecst staining of the nuclei. The fluorescent bright orange-green stain of Annexin V-FITC/PI in treated cells demonstrate apoptosis. The PI stain binds to the damaged nucleus of the treated cancer cells. In particular these image evidence that both TPC-1 and 8505C thyroid cancer cell lines are positive for annexin V after treatment with phenformin 1 mM, In addition thyroid cancer cell lines are positive for AnnexinV and PI after treatment with pjhenformin 10mM. On the other hand NHT cells results positive for AnnexinV/PI staining only after treatment with phenformin 10 mM, while are negative for treatment with phenformin 1mM.

The results of these cell viability experiments were confirmed by staining cells with Annexin V/PI, as shown in Figure 2. In details, both thyroid cancer cell lines, TPC-1 and 8505C, treated with 1 mM phenformin show Annexin V positive staining, which marks the exposure of phosphadidylserine at the outer leaflet with intact membrane integrity, but no PI fluorescence, suggesting an early stage of apoptotic events. NHT cells treated with the same concentrations of phenformin did not show Annexin V/PI staining, indicating that these cells were viable. The cells exposed to 10 mM phenformin, by contrast, are clearly positive for both Annexin V and PI fluorescence, which is indicative of loss of membrane integrity typical of a later apoptosis in all cell types.

The IC50 for treatment with phenformin at 24 hours was 7.4 mM for 8505C and 4.08 mM for TPC-1 cells, respectively. An IC50 could not be calculated for NHT cells as none of the tested concentrations elicited a 50% of reduction of cell viability.

### Basal secretion of the pro-tumorigenic chemokine CXCL8 after 7, 14 and 24 hours in NHT, TPC1 and 8505C cells.

**Figure 3:**
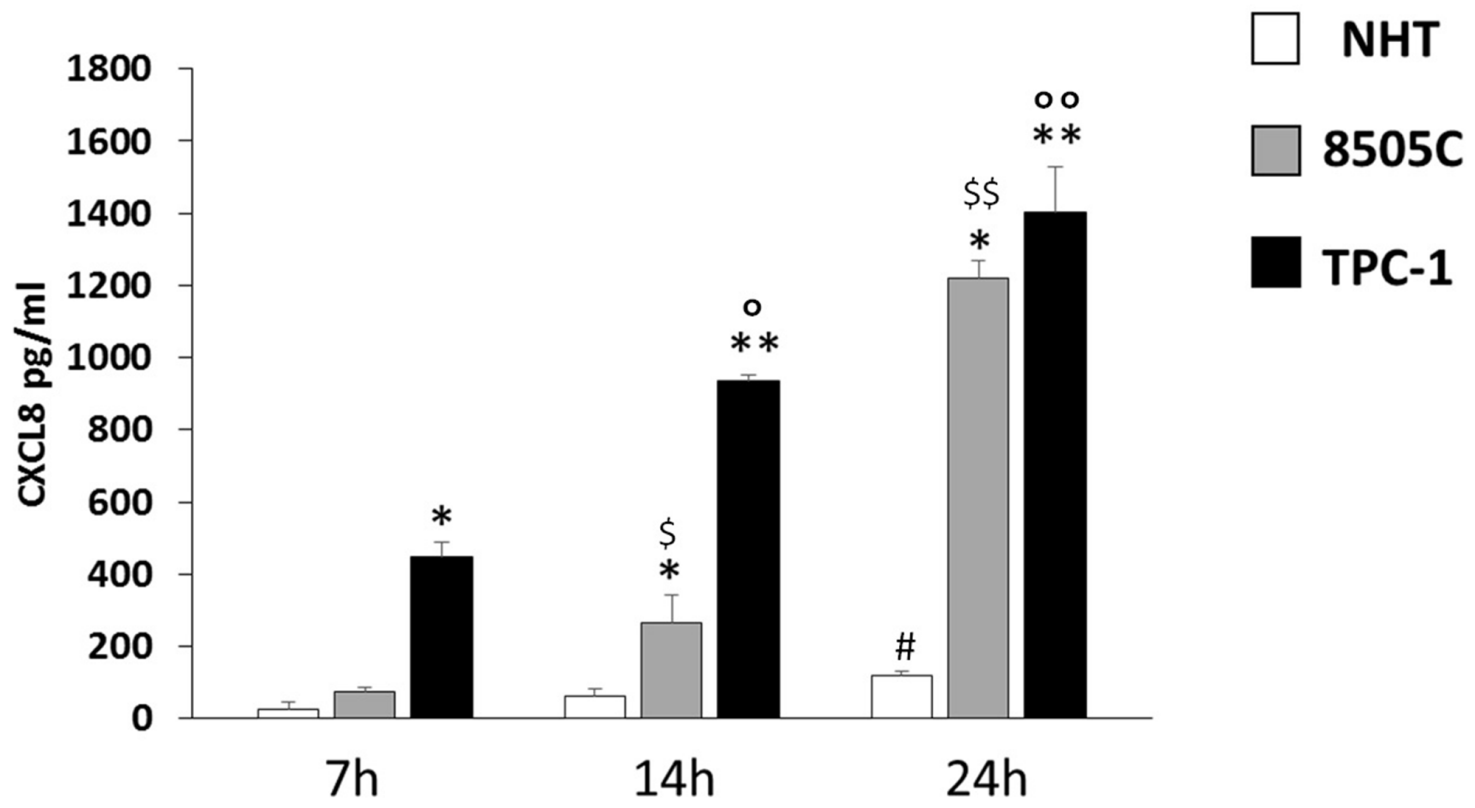
CXCL8 increase in NHT 8505C and TPC-1 throughout the time course NHT (ANOVA F=4.5 p<0.05; Post Hoc 24h p<0.05 vs. 7h), 8505C (ANOVA F=512.26 p<0.001; Post Hoc 24h p<0.05 vs. 14h and 7h, 14h p<0.05 vs. 7h), TPC-1 (ANOVA F=158.72 p<0.001; Post Hoc 24h p<0.05 vs. 14h and 7h, 14h p<0.05 vs. 7h). Panel D) the secretion of CXCL8 differs among the cell types at 7 14 and 24 hours being TPC-1>8505C>NHT. After a 7-hour incubation period CXCL8 levels were higher in TPC-1 supernatants as compared with the NHT and 8505C ones (ANOVA F=218.43 p<0.001; Post Hoc TPC-1 p<0.05 vs. 8505C and NHT). After 14 hours of incubation, TPC-1 cell again secreted the greatest amounts of CXCL8, followed by 8505C cells, which secreted higher levels as compared with NHT cells (TPC-1>8505C>NHT) (ANOVA F=332.78 p<0.001; Post Hoc TPC-1 p<0.05 vs. 8505C and NHT, 8505C p<0.05 vs. NHT). A similar secretion gradient was observed after 24 hours: TPC-1 > 8505C > NHT cells (ANOVA F=325.742 p<0.001; Post Hoc TPC-1 p<0.05 vs. 8505C and NHT, 8505C p<0.05 vs. NHT). ^*^p<0,05 vs. NHT; ^**^ p<0,05 vs. 8505C and NHT; #p<0,05 vs. 7h in NHT; $p<0,05 vs. 7h in 8505C; $$p<0,05 vs. 7h and 14h in 8505C; °p<0,05 vs. 7h in TPC-1; °°p<0,05 vs. 7h and 14h in TPC-1.

The levels of CXCL8 were measured in the supernatants from NHT, TPC-1, and 8505C cells in basal condition (Figure 3). The concentration of CXCL8 in the cell supernatant increased during the experiment time-course in NHT (ANOVA F=4.5 p<0.05; *Post Hoc* 24h p<0.05 *vs.* 7h), in 8505C (ANOVA F=512.26 p<0.001; *Post Hoc* 24h p<0.05 *vs.* 14h and 7h, 14h p<0.05 *vs.* 7h) and in TPC-1 (ANOVA F=158.72 p<0.001; *Post Hoc* 24h p<0.05 *vs.* 14h and 7h, 14h p<0.05 *vs.* 7h) cells, as shown in Figure 3. The absolute amounts of secreted CXCL8 greatly differed among normal and malignant cells. TPC-1 cells secreted the greatest amounts of CXCL8 while NHT produced the smallest ones. As shown in Figure 3, after a 7-hour incubation period CXCL8 levels were higher in TPC-1 supernatants as compared with the NHT and 8505C ones (ANOVA F=218.43 p<0.001; *Post Hoc* TPC-1 p<0.05 *vs.* 8505C and NHT). After 14 hours of incubation, TPC-1 cell again secreted the greatest amounts of CXCL8, followed by 8505C cells, which secreted higher levels as compared with NHT cells (TPC-1>8505C>NHT) (ANOVA F=332.78 p<0.001; *Post Hoc* TPC-1 p<0.05 *vs.* 8505C and NHT, 8505C p<0.05 *vs.* NHT). A similar secretion gradient was observed after 24 hours: TPC-1 > 8505C > NHT cells (ANOVA F=325.742 p<0.001; *Post Hoc* TPC-1 p<0.05 *vs.* 8505C and NHT, 8505C p<0.05 *vs.* NHT).

### Inhibiting effect of phenformin on CXCL8 secretion

**Figure 4:**
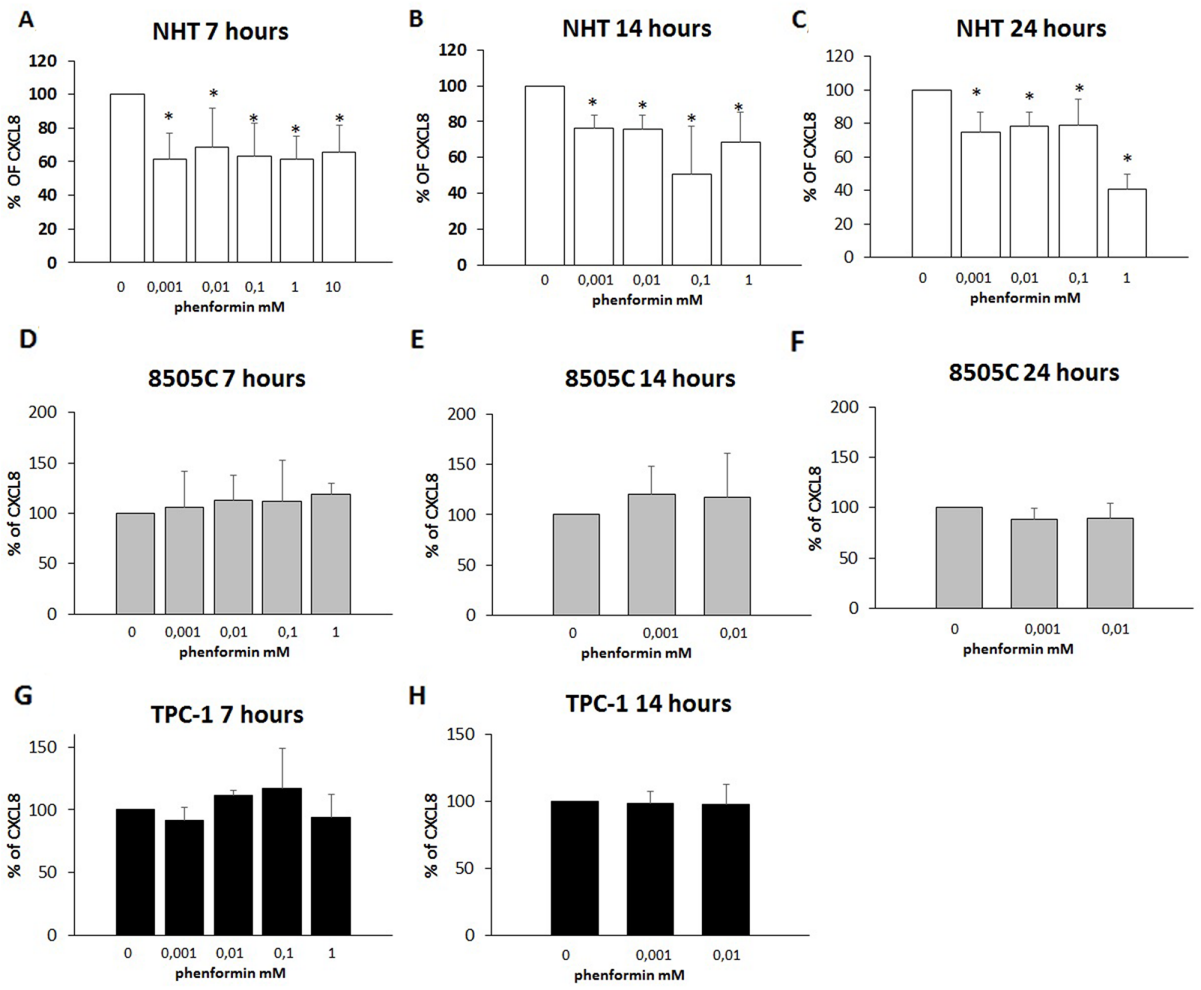
Inhibition of CXCL8 secretion **Panel A)** Phenformin inhibits CXCL8 secretion at all non-cytotoxic concentrations after 7 hour of treatment in NHT (ANOVA F=5.702; p<0.001) **Panel B)** Phenformin inhibits CXCL8 secretion at all non-cytotoxic concentrations after 14 hour of treatment in NHT (ANOVA F=11.15; p<0.001) **Panel C)** Phenformin inhibits CXCL8 secretion at all non-cytotoxic concentrations after 24 hours of treatment in NHT (ANOVA F=24.819; p<0.001). **Panel D)** Phenformin does not inhibit CXCL8 secretion after 7 hours of treatment in 8505C cells (ANOVA not significant) **Panel E)** Phenformin does not inhibit CXCL8 secretion after 14 hours of treatment in 8505C cells (ANOVA not significant) **Panel F)** Phenformin does not inhibit CXCL8 secretion after 14 hours of treatment in 8505C cells (ANOVA not significant) **Panel G)** Phenformin does not inhibit CXCL8 secretion after 7 hours of treatment in TPC-1 cells (ANOVA not significant) **Panel H)** Phenformin does not inhibit CXCL8 secretion after 14 hours of treatment in TPC-1 cells (ANOVA not significant) ^*^Post Hoc analysis by Bonferroni p<0.05 vs. basal.

The further step was to evaluate whether phenformin could exert any effect at concentrations lower than those required to induce cell death. To this aim, concentrations of phenformin not affecting cell viability at each time were tested for their ability to modulate the secretion of CXCL8 in TPC-1, 8505C and NHT cells. CXCL8 concentrations were assayed in the supernatants of, NHT, TPC-1 and 8505C at different times and phenformin concentrations. Phenformin inhibited CXCL8 secretion after 7 (ANOVA F=5.702; p<0.001), 14 (ANOVA F=11.15; p<0.001) and 24 hours (ANOVA F=24.819; p<0.001) in NHT (Figure 4 Panel A-B-C) cells. No inhibition of CXCL8 secretion was observed in TPC-1 and 8505C cells after 7 (TPC-1: ANOVA: F=1.434 NS; 8505C: ANOVA: F=1.161; NS), 14 (TPC-1: ANOVA: F=0.48 NS; 8505C: ANOVA: F=0.512 NS) and 24 hours of treatment with phenformin at concentrations not affecting cell viability (8505C: ANOVA: F=1.53 NS) (Figure 4 Panel D-H).

### Effects of phenformin on the ability of TPC-1 and 8505C to form colonies

**Figure 5:**
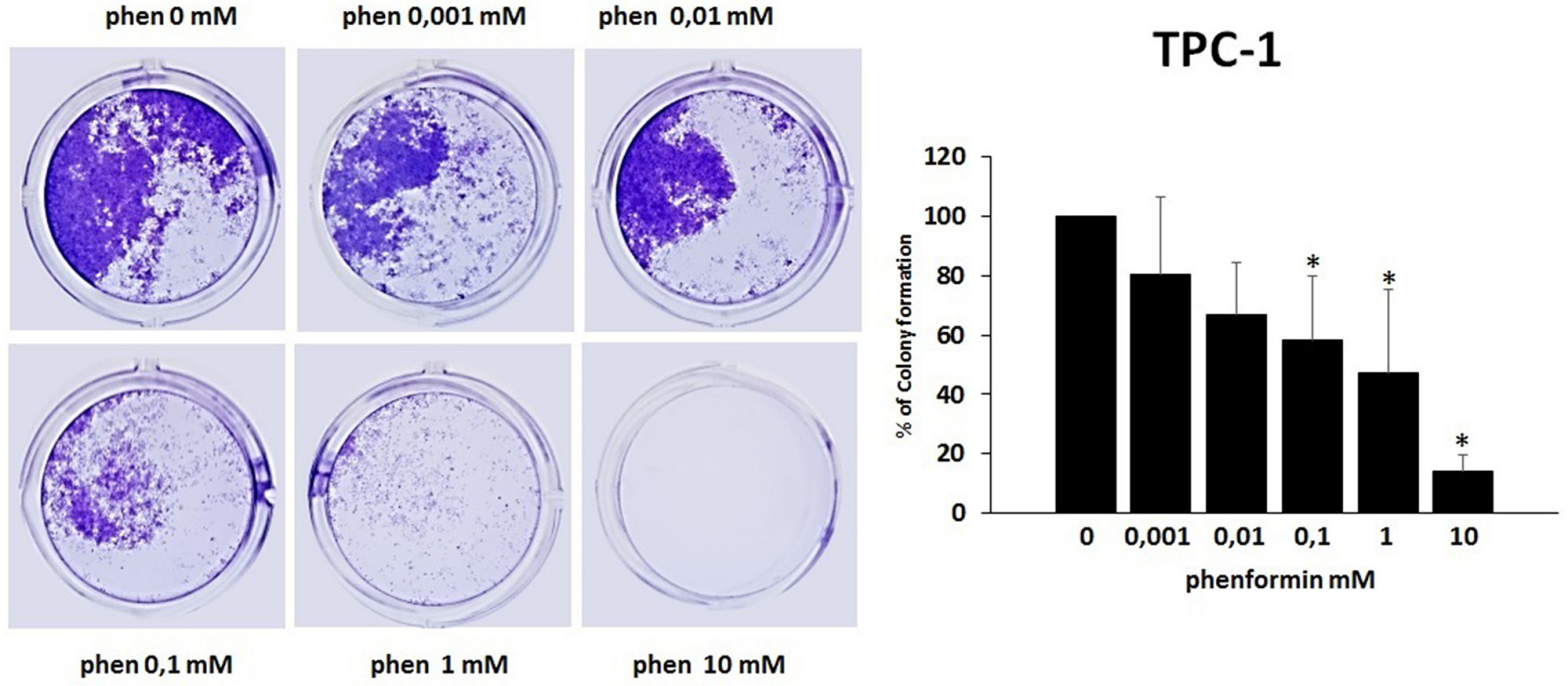
Effects of phenformin on TPC-1 cells ability to form colonies Colony formation assay was used to determine the long-term effects of phenformin on cell proliferation. The picture is one representative experiment of the results obtained. As indicated by histogram colony formation is reduced by phenformin treatment (ANOVA F=9.068 p< 0.0001) starting from 0.1mM concentration, as assessed by optical density measurement after staining with Crystal violet and incubation with SDS. Colony formation is expressed as a percentage of the untreated control. Post hoc ^*^p < 0.05, statistically significant.

**Figure 6:**
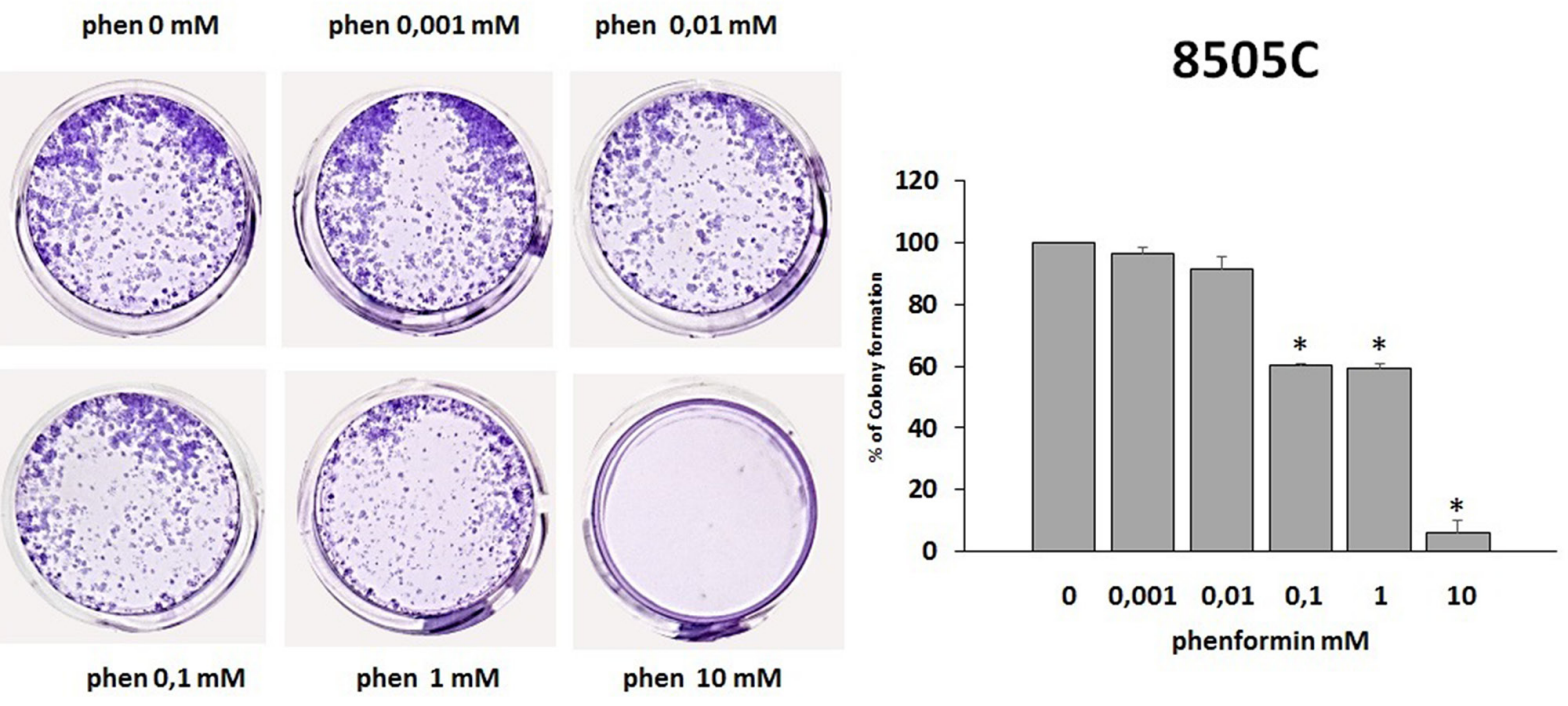
Effects of phenformin on 8505C cells ability to form colonies Colony formation assay was used to determine the long-term effects of phenformin on cell proliferation. The picture is one representative experiment of the results obtained. As indicated by histogram colony formation is reduced by phenformin treatment ANOVA F=311.75 p<0.0001) starting from 0.1mM concentration, as assessed by optical density measurement after staining with Crystal violet and incubation with SDS. Colony formation is expressed as a percentage of the untreated control. Post hoc ^*^p < 0.05, statistically significant.

The colony formation assay detects cells that have retained the capacity to produce a large number of progeny after pharmacological treatment. As shown in Figure 5 and Figure 6, treatment with phenformin at increasing concentrations reduced in a dose dependent manner the ability of both TPC-1 and 8505C to form colonies. Indeed, the percentage of colony formation was reduced by phenformin starting from the concentration of 0.1 mM in both cell types (ANOVA for TPC-1 F=9.06 p<0.0001 and ANOVA for 8505C F=311.75 p<0.0001) (Figure 5–6).

## DISCUSSION

Our study demonstrates, for the first time, that phenformin affects thyroid cells viability. Thyroid cancer cells are more vulnerable to the cytotoxic effect of phenformin than NHT cells. The phenformin-induced reduction of thyroid cancer cell viability occurs in a time- and dose-dependent manner. Indeed, in thyroid cancer cell lines a reduction of the viability was already observed after 7 hours of treatment with 10 mM phenformin, while a progressive decrease in the concentrations required to produce the effect was observed throughout the time-course. In contrast with tumor cells, a reduction of NHT cell viability could be observed only after prolonged time of treatment and with maximal doses of phenformin. These results indicate that normal and thyroid cancer cells profoundly differ in their response to phenformin in terms of cell death induction.

Furthermore the effect of phenformin on cell viability was confirmed by AnnexinV/PI staining which also suggests the involvement of an apoptotic pathway induced by the drug exclusively in thyroid cancer cell lines and not in NHT.

The fact that the viability of normal thyroid cells was not affected by phenformin (unless when a very high concentration was used) suggests that these cells could be preserved during an hypothetical anti-cancer treatment with phenformin, being thyroid cancer cells the only ones profoundly and selectively damaged by the drug.

The experimental design of the present study does not allow drawing conclusions on the mechanisms through which phenformin reduces cell viability in thyroid cancer cells, nor on the reason why normal thyroid cells are preserved by this cytotoxic effect. However, it could be speculated that this effect would rely upon the rate of cellular replication, reducing the viability mainly on the more rapidly replicating cells (thyroid cancer cell lines). The above statement would be supported by the fact that the reduction of cell viability exerted by phenformin was related to the specific cell proliferation rate. Indeed, it was previously reported that higher proliferation rate characterizes TPC-1 cells as compared to 8505C [29] as assessed by higher incorporation of BrdU (which indicates the DNA replication). Furthermore the proliferation rate of NHT primary cells is clearly lower when compared with that of thyroid cancer cell lines [30]. In the present study, these differences in the proliferation rate were paralleled by a strong (TPC-1), intermediate (8505C) and weak (NHT) cytotoxic effect of phenformin. These results suggest that phenformin behaves like a classic cytotoxic chemotherapy agent, which primarily affects rapidly proliferating cells [31].

A second finding of the present study is that phenformin, at non-cytotoxic concentrations, does inhibit the secretion of CXCL8 in normal thyroid cells only. Experimental data from previous *in vitro* and *in vivo* studies indicate that CXCL8 is responsible for a more aggressive clinical course of differentiated thyroid cancer [25, 32, 33]. CXCL8 favors tumor progression by promoting the metastatic spread of thyroid cancer through the induction of epithelial to mesenchymal transition [34, 35]. As a matter of fact, targeting the receptors for CXCL8 proved to have therapeutic benefits in experimental models of thyroid cancer [33, 36]. Thus, in addition to a direct anti-tumor effect (induction of cell death), phenformin also exerts indirect anti-tumor effects through a modification of the chemokine milieu within the tumor microenvironment (e.g. inhibition of CXCL8 secretion). Several agents were previously reported to have an inhibiting effect on the secretion of CXCL8 in thyroid cancer microenvironment. These include AICAR, PLX4720, Interferons and also the biguanide metformin [25, 27, 37–42]. The finding that phenformin inhibits the CXCL8 secretion only in NHT but not in thyroid cancer cells should be briefly discussed. Literature data, evidence that the specific genetic mutation harbored by thyroid cancer cells is related to a different ability to secrete CXCL8, which generally occurs at a higher level than NHT [25, 43–46]. The results of the present study showed that the CXCL8 basal levels as well as those reached throughout the time course, were higher in TPC-1 than in 8505C and lowest in NHT cells. This is in line with the notion that the secretion of CXCL8 by thyroid cells is dependent upon different pathways which are switched on by specific genetic lesions more or less effective in enhancing the secretion of CXCL8 [43]. Therefore, since the mechanisms of CXCL8 secretion differ among cell types, it appears not surprising that also pharmacological inhibiting strategies would be different.

The results of the present study indicate that phenformin has different effects in thyroid cancer cell, in which it affects viability, and in NHT cells, in which it inhibits the secretion of CXCL8. The latter effect is particularly relevant because NHT cells are the most represented ones in thyroid cancer microenvironment. This dual effect of phenformin underlines its peculiar anti-cancer properties and would also suggest multiple benefits in reducing tumor progression.

The observation that phenformin reduces the ability of thyroid cancer cells to form colonies expands the potential benefits of this molecule as an anti-cancer drug in vivo. This is because the eradication of cancer cells with unlimited proliferating ability is required for the prevention of recurrences [47]. In this scenario, phenformin would not only affect thyroid cancer cell viability, but would also limit their ability to form colonies. Taken together, our data provide evidence for multiple anti-cancer properties of phenformin in thyroid cancer.

Our findings are particularly relevant because phenformin is a relatively low toxicity drug, at least as compared with many commonly used chemotherapeutic agents [48]. The main side effect of phenformin is the possible induction of lactic acidosis [5], which, however, is prevented by supplementation with 2-deoxyglucose [18]. Despite the here reported *in vitro* lack of cytotoxicity of phenformin in NHT, the possibility of potential toxic effects of phenformin cannot be ruled out. In this view, an ongoing clinical trial (NCT03026517), evaluating the potential toxicity of increasing doses of phenformin in combination with Dabrafenib and Trametinib in patients with melanoma, will provide useful information about the safety profile of phenformin in humans.

In conclusion, the results of our *in vitro* study support the idea that phenformin might be used as an anti-tumor drug for the treatment of thyroid cancer. Moreover, as previously shown in melanoma, phenformin could also exert a synergic effect with other anti-cancer drugs, such as immune-therapeutic agents or chemotherapeutic compounds. Future mechanistic studies, as well clinical trials, are required to further understand the complex role of phenformin as an anti-cancer therapeutic agent.

## MATERIALS AND METHODS

### Primary cultures of normal human thyroid (NHT) cells

Surgical specimens of normal human thyroid were obtained from the contralateral disease-free lobe of patients who underwent thyroidectomy for a solitary non-functioning nodule (n=3). The study was approved by the Institutional Review Board of ICS-Maugeri. Before surgery, written informed consent for the study was obtained from all patients. All experiments were performed in accordance with the relevant guidelines and regulations. Surgical specimens were minced and then incubated with collagenase type II (Sigma, Saint Louis, MO, USA) 5 mg/ml, in 5 ml of Coon’s F12 medium, for 4 hours at 37°C. Then, 10 ml of Coon’s F12 medium were added, following which, cells were filtered, spun at 1000 × g for 10 min, washed with Coon’s F12 medium, spun again, and finally re-suspended in complete medium containing 5% newborn calf serum and a mixture of six hormones including insulin (5μg/ml), hydrocortisone (50 μg/ml), transferrin (5 μg/ml), somatostatin (10 ng/ml), gly-his-lysine (10 ng/ml) and bovine TSH (1 mU/ml).

### Thyroid tumor cell lines 8505C and TPC-1

The human thyroid cancer cell lines TPC-1, bearing the RET/PTC rearrangement, and 8505C, harboring the BRAF V600E mutation, had been previously tested and authenticated by DNA analysis. Cancer cells were propagated in Dulbecco’s Modified Eagle Medium (DMEM) and RPMI (Sigma, Saint Louis, MO, USA) supplemented with 10% fetal bovine serum (Sigma, Saint Louis, MO, USA), 2mM L-glutamine and 100 U/ml penicillin/streptomycin (Sigma, Saint Louis, MO, USA). Cells were incubated with the chosen stimuli in serum-free medium.

### Cell viability, WST-1 assay

NHT, TPC-1 and 8505C were grown in complete medium until an 80% confluence was reached. Cells were then detached and seeded in 96 well flat plates at a density of 2×10^4^cell/well. Complete medium was supplemented with increasing concentrations of phenformin (0, 0.001, 0.01, 0.1, 1, 10 mM, Sigma Aldrich), the concentrations of phenformin were chosen based on previous studies [7, 49, 50]. The incubation times were 7, 14 and 24 hours. At the end of treatment, 20 µl of WST-1 were added to wells; plates were then incubated for 30 minutes at 37°C in a 5% CO_2_ atmosphere. WST-1 is a colorimetric reagent, which, after cleavage of a tetrazolium salt, MTS, by mitochondrial dehydrogenases, results in the production of formazan by viable cells only. Absorbance was then measured at 450 nm by using a multimode plate reader (Victor NIVO Multimode Plate Reader, PerkinElmer). All experiments were performed in triplicates. Values of IC50s (concentrations necessary to reduce the cell viability by 50%) were calculated for the various cell lines from concentration-response curves by linear regression analysis (percent/inhibition against the negative natural logarithm of the molar concentration of phenformin).

### Annexin V-FITC/PI assay to detect cell death

The cell surface exposure of phosphatidylserine on the plasma membrane of cells were assessed using Annexin V-FITC Apoptosis Detection Kit (Life-Technologies Apo-Detect Kit). Briefly, cells were harvested on a coverslip sited in a 24-well plate at a density of 10^4^ cells per well. After adhesion, cells were treated with phenformin at 0, 1 and 10 mM. After 24 hours of treatment cells were washed with PBS and supernatants were conserved for cyto-spin. Coverslips were incubated with a mix of Annexin V-FITC, PI and Hoechst 33258 (Thermofisher) in the dark for 10 min at room temperature. The same procedure was reserved for cells recovered in the supernatants after centrifugation. Cells were fixed with PFA 4% for 10 minutes. After washing with PBS, coverslips were mounted with Dako and the fluorescence images were obtained with a Leica TCS-SP5 II inverted confocal microscope.

### CXCL8 secretion by NHT cells, TPC-1 and 8505C thyroid cancer cell lines and NHT cells in the presence or absence of increasing concentrations of phenformin.

For the CXCL8 secretion assays, 3000 cells were seeded into 96-well plates in complete medium. After adherence to the plastic surface, NHT, TPC-1 and 8505C cells were incubated in serum-free medium with or without (basal condition) increasing non-cytotoxic concentrations of phenformin (Sigma Aldrich) for 7-14 and 24 hours. All experiments were performed in triplicates.

### ELISA for CXCL8

CXCL8 was measured in cell supernatants of NHT, TPC-1, and 8505C and cells using commercially available kits (R&D Systems, Minneapolis, MN). The mean minimum detectable concentration of CXCL8 was 3.5 pg/ml. The intra- and inter-assay coefficients of variation were 3.4% and 6.8%, respectively. Samples were assayed in duplicates.

### Colony formation assay

Colony formation assay is an *in vitro* cell survival assay that tests the ability of a single cancer cell to undergo ‘‘unlimited’’ division, thus growing into a colony. This is the method of choice to determine the loss of cancer cells replicating capacity after treatment with a given compound [47].

TPC-1 and 8505C cell lines were incubated in the presence or absence of increasing concentrations of phenformin for 24 hours. Cells were detached with trypsin, plated into 24-well plates (2 × 10^3^ cells/well) and maintained in complete medium for 8 days. Cells were fixed with methanol for 20 minutes and stained with 0.5% crystal violet dye for 5 minutes [51]. Colony formation was confirmed under an inverted light microscope Olympus BX51 microscope (Olympus, Deutschland GmbH, Hamburg, Germany). For quantification, after three washes with deionized water to remove excess stain, the crystal violet dye was released from the cells by incubation with 1% SDS for 2 hours before optical density (OD) 570 nm measurement.

### Statistical analysis

Statistical analysis was performed using the SPSS software (SPSS, Inc., Evanston, IL). Mean group values were compared by using one-way ANOVA for normally distributed variables. Post hoc analysis was performed according to the Bonferroni’s correction for multiple comparisons. Values are reported as mean ± SD unless otherwise noted. A *p* value < 0.05 was considered statistically significant.

## Abbreviations

CXCL: chemokine (C-X-C motif) ligand
NHT: normal human thyreocytes
PI: propidium iodide
PLX4720: Plexxikon compound 7-azaindole derivative
PTC: papillary thyroid carcinoma
RAI: radioactive iodine
TSH: thyroid stimulating hormone
